# *Tsc1* Haploinsufficiency Leads to Pax2 Dysregulation in the Developing Murine Cerebellum

**DOI:** 10.3389/fnmol.2022.831687

**Published:** 2022-05-13

**Authors:** Ines Serra, Ana Stravs, Catarina Osório, Maria Roa Oyaga, Martijn Schonewille, Christian Tudorache, Aleksandra Badura

**Affiliations:** ^1^Department of Neuroscience, Erasmus MC, Rotterdam, Netherlands; ^2^Institute of Biology Leiden, Leiden University, Leiden, Netherlands

**Keywords:** tuberous sclerosis complex (TSC), mouse model, autism spectrum disorder (ASD), cerebellar development, Pax2

## Abstract

Tuberous sclerosis complex 1 (TSC1) is a tumor suppressor that promotes the inhibition of mechanistic target of rapamycin (mTOR) pathway, and mutations in *TSC1* lead to a rare complex disorder of the same name. Despite phenotype heterogeneity, up to 50% of TSC patients present with autism spectrum disorder (ASD). Consequently, TSC models are often used to probe molecular and behavioral mechanisms of ASD development. Amongst the different brain areas proposed to play a role in the development of ASD, the cerebellum is commonly reported to be altered, and cerebellar-specific deletion of *Tsc1* in mice is sufficient to induce ASD-like phenotypes. However, despite these functional changes, whether *Tsc1* haploinsufficiency affects cerebellar development is still largely unknown. Given that the mTOR pathway is a master regulator of cell replication and migration, we hypothesized that dysregulation of this pathway would also disrupt the development of cell populations during critical periods of cerebellar development. Here, we used a mouse model of TSC to investigate gene and protein expression during embryonic and early postnatal periods of cerebellar development. We found that, at E18 and P7, mRNA levels of the cerebellar inhibitory interneuron marker paired box gene 2 (*Pax2*) were dysregulated. This dysregulation was accompanied by changes in the expression of mTOR pathway-related genes and downstream phosphorylation of S6. Differential gene correlation analysis revealed dynamic changes in correlated gene pairs across development, with an overall loss of correlation between mTOR- and cerebellar-related genes in *Tsc1* mutants compared to controls. We corroborated the genetic findings by characterizing the mTOR pathway and cerebellar development on protein and cellular levels with Western blot and immunohistochemistry. We found that Pax2-expressing cells were largely unchanged at E18 and P1, while at P7, their number was increased and maturation into parvalbumin-expressing cells delayed. Our findings indicate that, in mice, *Tsc1* haploinsufficiency leads to altered cerebellar development and that cerebellar interneuron precursors are particularly susceptible to mTOR pathway dysregulation.

## Introduction

The mechanistic target of rapamycin (mTOR) pathway is a highly complex, conserved and ubiquitous signaling pathway involved in biomass synthesis, growth and cell proliferation (Liu and Sabatini, 2020). Specifically, it has been proposed that a tight regulation of mTOR signaling is required for sustaining cell cycle length and re-entry, defining pluripotency status and triggering differentiation during brain development (Easley et al., 2010; Hartman et al., 2013; Ka et al., 2014). However, how the mTOR pathway affects distinct lineages of differentiating cells is still largely unexplored. Supporting a crucial role for mTOR signaling in brain development, mutations in this pathway frequently lead to complex monogenic neurodevelopmental disorders (also known as *mTORopathies*), characterized by heterogeneous neuropsychiatric phenotypes that include megalencephaly, epilepsy, intellectual disability and autism spectrum disorder (ASD) (Moloney et al., 2021; Nguyen and Bordey, 2021).

The prototypical mTORopathy is tuberous sclerosis complex (TSC), a rare autosomal dominant disorder affecting 1 in 6000 people, that arises from heterozygous mutations in the *TSC1* or *TSC2* genes (Haines et al., 1991; Northrup et al., 1992). As TSC1 and 2, together with TBC1D7, form a tumor suppressor complex upstream of mTOR, loss of function of this complex leads to mTOR pathway overactivity (Crino, 2013; Qin et al., 2016). mTOR can be organized in two complexes, mTORC1 and mTORC2, characterized, among others, by the presence of Raptor and Rictor, respectively (Kim et al., 2002; Sarbassov et al., 2004). While mTORC1 is primarily associated with growth and proliferation, mTORC2 regulates cytoskeleton organization and cell motility (Masri et al., 2007; Hoxhaj et al., 2017). Nonetheless, crosstalk between mTORC1 and mTORC2 is vast, and changes in the function of both complexes due to mTOR dysfunction were shown to alter dendritic arbor morphology and synaptic transmission (McCabe et al., 2020). On the whole, the effects of mTOR overactivity in TSC patients lead to a multi-system phenotype that includes widespread hamartoma growth, high prevalence of epilepsy and, in up to 50% of the patients, ASD (Kingswood et al., 2017; McDonald et al., 2017).

Despite its high prevalence, little is known about the molecular mechanisms that underlie ASD, although a number of genetic and environmental risk factors have now been identified (Elsabbagh et al., 2012; Wang et al., 2014). While there is no single major anatomical abnormality evident in all people with ASD, the cerebellum is a brain structure that has emerged as a significant putative contributor to the development of ASD phenotypes. In humans, damage to the cerebellum is the second largest factor contributing to the risk of developing ASD (Limperopoulos et al., 2007; Wang et al., 2014; van der Heijden et al., 2021), while cerebello-cortical connectivity is often found impaired in people with ASD (Stoodley et al., 2017; Kelly et al., 2020). In recent years, several studies showed that murine models with cerebellar-specific deletion or inactivation of mTOR pathway genes present with ASD-like phenotypes, exhibiting decreased social interaction, increased repetitive behaviors and inflexible learning (Goorden et al., 2007; Tsai et al., 2012, 2018; Badura et al., 2018). Together with the fact that many mTOR pathway genes are found to be enriched in the cerebellum (Menashe et al., 2013; Li et al., 2018), this suggests that this brain area may be particularly sensitive to changes in mTOR pathway regulation. However, how disruption of the mTOR pathway affects cerebellar development is currently unknown.

Here, we used a haploinsufficient *Tsc1* mouse model to investigate the effects of Tsc1 deficiency in the developing cerebellum. We found that genetic dysregulation of the mTOR pathway can be detected from E18, suggesting a compensatory down-regulation in response to the hyperactivity of this pathway. Changes to cerebellar development can also be found at this age and postnatally at P7. Specifically, we found that paired box gene 2 (Pax2) expression at E18 is altered, indicative of a delay in its expression initiation in *Tsc1* mice. At P7, this culminated in reduced parvalbumin expression and interneuron maturation. Overall, our data suggest that mTOR overactivity in the cerebellum preferentially affects the development of cerebellar interneurons, which could potentially promote the development of altered circuitry and, consequently, lead to behavioral deficits.

## Materials and Methods

### Experimental Procedures

All experimental animal procedures were approved by an independent animal ethical committee (DEC-Consult, Soest, The Netherlands), as required by Dutch law, and conform to the relevant institutional regulations of the Erasmus MC and Dutch legislation on animal experimentation.

### Animals

Timed pregnancies were established between wild-type C57BL/6 females (*Tsc1*^+/+^) (Charles River Laboratories; *n* = 22) and *Tsc1*^tm 1Djk^ (*Tsc1*^+/–^) males to obtain mixed *Tsc1*^+/+^ and *Tsc1*^+/–^ litters (Kwiatkowski et al., 2002). Vaginal plugs were checked daily, and embryonic day 0 (E0) was defined when a plug was observed. Confirmed pregnant dams were individually housed. Mice were maintained on a standard 12h light/dark cycle, with access to food and water *ad libitum*.

In total we used: *n* = 16 E15 embryos (*n* = 8 mice per genotype), *n* = 30 E18 embryos (*n* = 15 mice per genotype), *n* = 34 P1 pups (*n* = 17 mice per genotype), and *n* = 36 P7 pups (*n* = 18 mice per genotype).

### Real-Time Quantitative Polymerase Chain Reaction

#### Primer Design

Seven genes of interest along the TSC-mTOR pathway (*Tsc1*, *Tsc2*, *Rictor*, *Rptor*, *Mtor*, *Rps6kb1*, and *Rps6*) (Liu and Sabatini, 2020) and 5 genes representing distinct cerebellar lineages (*Pax2*, *Pax6*, *Calb1*, *Slc1a3*, and *Gdf10*) (Carter et al., 2018) were targeted. Housekeeping genes were selected based on previous literature using embryonic and neonatal mouse brain tissue (Cheung et al., 2017; Xu et al., 2017; Shaydurov et al., 2018). Two housekeeping genes were selected per age: *Ywhaz* and *Sdha* were used for the E15 group, *Gusb* and *Sdha* for E18, and *Gusb* and *Ywhaz* for P1 and P7.

Primer pairs were adapted from literature or designed using *Primer-BLAST*^1^ and *Ensembl* (m.ensembl.org) (Table 1).

**TABLE 1:** Primer sequences. Overview of genes and primer pairs used for quantitative real time PCR and genotyping of WT and *Tsc1*^+/–^ mice.

Targeted gene	Primer: forward sequence	Primer: reverse sequence
**qPCR**		
*Calb1*	TCTGGCTTCATTTCGACGCTG	ACAAAGGATTTCATTTCCGGTGA
*Gdf10*	CAGGACATGGTCGCTATCCAC	ACAGGCTTTTGGTCGATCATTTC
*Gusb*	CACACTGACCCCTCATACCC	TGCAGTCCCGCATAGTTGAA
*Mtor*	CACCAGAATTGGCAGATTTGC	CTTGGACGCCATTTCCATGAC
*Pax2*	AAGCCCGGAGTGATTGGTG	CAGGCGAACATAGTCGGGTT
*Pax6*	TACCAGTGTCTACCAGCCAAT	TGCACGAGTATGAGGAGGTCT
*Rptor*	CAGTCGCCTCTTATGGGACTC	GGAGCCTTCGATTTTCTCACA
*Rictor*	ACAGTTGGAAAAGTGGCACAA	GCGACGAACGTAGTTATCACCA
*Rps6kb1*	AGCCCTGATGACTCCACTCT	CTGACAGGTGTTCGTGGACT
*Rsp6*	CTGGGTTAAGCGGAAGTCGG	CCACCTCGATGAGCTTCTGA
*Sdha*	GGAACACTCCAAAAACAGACCT	CCACCACTGGGTATTGAGTAGAA
*Slc1a3*	CCGACCGTATAAAATGAGCTACC	ATTCCTGTGACGAGACTGGAG
*Tsc1*	CGGCTCTGGAGGAACACAAT	GCTGACTGTATCGGGCTTGT
*Tsc2*	AGTTCTCACCTTATTGAAGGCCA	CATTGGAGGGGTAGTCCTTGA
*Ywhaz*	GAAAAGTTCTTGATCCCCAATGC	TGTGACTGGTCCACAATTCCTT
**Genotyping**		
*Tsc1*	GTCACGACCGTAGGAGAAGC AGGAGGCCTCTTCTGCTACC	GAATCAACCCCACAGAGCAT

Primers were validated for their specificity *in silico* using *UCSC in silico PCR*,^2^ *PrimerBank*^3^ and *blastn*^4^, and *in vitro* with conventional PCR and melt curve analysis.

#### RNA Extraction

For the collection of embryonic samples, pregnant dams were briefly anesthetized prior to cervical dislocation, and E15 (*n* = 8 mice per genotype) and E18 (*n* = 6 mice per genotype) embryos collected onto cold PBS on ice. For the collection of neonatal samples, P1 (*n* = 8 mice per genotype) and P7 (*n* = 8 mice per genotype) mice were anesthetized prior to decapitation. Cerebellar tissue was dissected in cold PBS under a Zeiss Stemi SV6 Stereo microscope, collected into TRI Reagent^®^ (T9424, Sigma-Aldrich, MO, USA) and kept at −80°C until used.

Following dissection, RNA was isolated using a standard chloroform/isopropanol method (Rio et al., 2010). In brief, tissue in TRI Reagent^®^ was thawed, homogenized by syringe aspiration (G23 and G25) and vortexed. Chloroform (1:5) was added to the sample, followed by a 5-minute incubation at room temperature (RT). Samples were then centrifuged at 10,800 *g*, at 4°C for 15 min. The aqueous phase was collected and a 1:1 ratio of isopropanol was added. Samples were centrifuged for 10 min at maximum speed (∼20 000 *g*). The obtained RNA pellet was washed twice with 70% ethanol, air-dried, resuspended in 20 μL of RNase-free water (UltraPure*™* DNase/RNase-Free Distilled Water, 10977-035, Invitrogen, MA, USA), and quantified with NanoDrop (Thermo Fisher Scientific, MA, USA).

#### Real-Time-Quantitative Polymerase Chain Reaction

RNA was transcribed using qScript^®^ cDNA SuperMix (Quantabio, MA, USA, 95048-100), according to manufacturer’s instructions. RT-qPCR was performed with PerfeCTa^®^ SYBR^®^ Green FastMix^®^ (Quantabio, MA, USA, 95072-05K) following manufacturer’s instructions, with 10 μM of forward and reverse primers, and 1 μL cDNA (diluted 1:5). All samples were run in duplicate. RT-qPCR was performed in a CFX96*™* Real-Time PCR detection system (Bio-Rad Laboratories, Inc., CA, USA), with initial denaturation for 1 min at 95°C, followed by 40 cycles of 5 s at 95°C, and 15 s at 55°C, with melting curve generation.

### Raw Data Processing

Relative quantification was performed as in Taylor et al. (2019), on 8 biological samples per genotype for the E15, P1 and P7 groups, and on 6 biological samples for the E18 group. The mean quantitative cycle (Cq) values were extracted for each sample (genes of interest and housekeeping genes), and the mean Cq per gene was calculated within the control group (WT). A ΔCq was then calculated per sample by subtracting the control group average from the sample mean Cq. For each sample, the relative quantities were then calculated [(1 + E)^ΔCq^, *E* = 1]. Normalized expression per sample (genes of interest) was obtained by dividing the relative quantity of a given sample by the geometric mean of the relative quantities of the two housekeeping genes. The average normalized expression of the samples, in each genotype per gene of interest, was calculated, and the geometric mean with SEM error bars plotted.

### Western Blot

Cerebellar tissue from E18 (*n* = 4 mice per genotype), P1 (*n* = 4 mice per genotype) and P7 (*n* = 5 mice per genotype), WT and *Tsc1*^+/–^ mice, was dissected and immediately frozen in dry ice. Samples were homogenized with a Dounce homogenizer in ice-cold lysis buffer containing 50 mM Tris–HCl pH 8, 150 mM NaCl, 1% Triton X-100, 0.5% sodium deoxycholate, 0.1% SDS and protease inhibitor cocktail (Roche, Basel, Switzerland). Protein concentrations were measured using a Pierce BCA protein assay kit (Thermo Fisher Scientific, MA, USA). Samples were denatured and proteins separated in SDS-PAGE in Criterion*™* TGX Stain-Free*™* Gels (Bio-Rad Laboratories, Inc., CA, USA), and transferred onto nitrocellulose membranes with the Trans-Blot^®^ Turbo*™* Blotting System (Bio-Rad Laboratories, Inc., CA, USA).

Membranes were blocked with 5% BSA (Sigma-Aldrich, MO, USA) in Tris-buffered saline (TBS)-Tween (20 mM Tris–HCl pH7.5, 150 mM NaCl and 0.1%, Tween 20) for 1 h, and probed with the following primary antibodies: Pax2 (1:1000, rabbit, Cell Signaling Technology Inc., MA, USA, 9666), Phospho-S6 Ribosomal Protein (Ser235/236) (1:1000, rabbit, Cell Signaling, Technology Inc., MA, USA, 2211), Ribosomal Protein S6 (1:1000, mouse, Santa Cruz, Biotechnology, TX, USA, SC-74459) or GAPDH (1:1000, mouse, Cell Signaling, Technology Inc., MA, USA, 97166). Secondary antibodies used were goat anti-Rabbit Immunoglobulins/HRP (1:10000, Agilent Dako, CA, USA, P0448) or goat anti-Mouse Immunoglobulins/HRP (1:10000, Agilent Dako, CA, USA, P0447). Proteins were detected by the luminol-based enhanced chemiluminescence method (SuperSignal*™* West Femto Maximum Sensitivity Substrate or SuperSignal*™* West Dura Extended Duration Substrate, Thermo Fisher Scientific, MA, USA). Membranes were stripped with Restore*™* PLUS Western Blot Stripping Buffer (Thermo Fisher Scientific, MA, USA). Densitometry of protein bands of interest was normalized to that of GAPDH using the Image Studio Lite software (LI-COR Biosciences, NA, USA), and protein expression normalized to WT values plotted.

### Immunohistochemistry

After collection, E18 embryos (*n* = 5 mice per genotype) were fixed by immersion in cold 4% paraformaldehyde (PFA) in phosphate buffered saline (PBS). P1 (*n* = 5 per genotype) and P7 pups (*n* = 5 WT and 4 *Tsc1*^+/–^ mice) were injected with an overdose of pentobarbital and transcardially perfused with 4% PFA in PBS. Afterwards, tissue was placed in 4% PFA for 2 h and transferred into 30% sucrose in 0.1 M phosphate buffer (PB) until embedding. Samples were embedded in 14% gelatin/30% sucrose and incubated in a 10% PFA/30% sucrose solution for 1.5 h, at RT, on a shaker. Embedded samples were kept in 30% sucrose/0.1 M PB at 4°C until cut.

Cerebellar samples were cut in 30 μm sagittal sections using a cryomicrotome (Leica SM 200R). Free-floating sections were rinsed with PBS and preincubated with 10% normal horse serum (NHS)/0.5% Triton*™* X-100 in PBS, for 1 h at RT on a shaker. Sections were then incubated overnight, at 4°C, in 2% NHS/0.4% Triton*™* X-100 in PBS with primary antibodies against Pax2 (1:500, rabbit, Invitrogen, MA, USA, 71-6000 or 1:500, goat, R&D Systems, MN, USA, AF3364), Parvalbumin (1:500, mouse, Swant 235), phosphorylated S6 (Ser235/236) (1:500, rabbit, Cell Signaling Technology Inc., MA, USA, 2211) or Calbindin (1:10.000, mouse, Sigma-Aldrich, MO, USA, C9848). The following day, sections were rinsed with PBS and incubated with AlexaFluor 594 (1:1000, Donkey anti-rabbit, Jackson Immuno Research Labs, PA, USA, 711-585-152), AlexaFluor 647 (1:1000, Donkey anti-goat, Jackson Immuno Research Labs, PA, USA, 705-605-003), AlexaFluor 488 (1:1000, Donkey anti-mouse, Jackson Immuno Research Labs, PA, USA, 715-545-150) or Cy3 (1:1000, Donkey anti-rabbit, Jackson Immuno Research Labs, PA, USA, 711-165-152) in 2% NHS/0.4% Triton*™* X-100 in PBS, for 1.5 h at RT. Sections were rinsed, counterstained with DAPI (1:10.000), and rinsed again with PB before mounting. Sections used for data analysis were imaged with a 10X (E18) or 20X (P1 and P7) objective using a Zeiss AxioImager.M2 microscope (representative images were taken using Zeiss AxioImager.M2 with Apotome 3).

### Microscopy Image Quantification

The number and area of Pax2^+^ positive cells were automatically counted with Fiji ImageJ (Schindelin et al., 2012), using custom-written macros.^5^ Given the positive correlation between nuclear and cell body size, we used the area of Pax2^+^ staining as a proxy for cell area (Wu et al., 2022). To calculate the distance between Pax2^+^ particles, we used the ND ImageJ plugin (Haeri and Haeri, 2015).

Calbindin-stained sections from P7 WT and *Tsc1*^+/–^ mice were used to measure the area of Purkinje cells. The area of 10 randomly selected Purkinje cells per mouse, located between lobules V and VI, was manually measured with Fiji ImageJ, by drawing a region of interest around its visible cell body cross-section.

At P7, parvalbumin (PV) staining was still sparse and dispersed in the developing cerebellum. Thus, we opted for the measurement of the total area occupied by PV stain rather than counting individual cells. To do this, PV-stained cerebellar sections were automatically thresholded and a region of interest was defined including the whole cerebellar section while excluding the already existing Purkinje cell layer (see *Results*). This enabled the measurement of the PV-signal primarily derived from developing molecular layer interneurons (Simat et al., 2007).

### Statistics

All statistical analysis was performed on GraphPad Prism 8. Data were first screened for the presence of outliers using the ROUT method, and tested for normality using the Shapiro–Wilk test, when applicable. When the normality assumption was followed, a two-tailed *t*-test was used for data comparison. When this assumption was violated, a two-tailed Mann-Whitney test was used. Variable correlation was performed using Pearson’s correlation, and simple linear regression was used for line fitting.

## Results

### mTOR Pathway and Cerebellar Cell Type-Specific Gene Transcription Is Dysregulated in *Tsc1*^+/–^ Cerebella

To first investigate whether *Tsc1* haploinsufficiency changes the expression of a number of genes relevant to the mTOR pathway and cerebellar development, we performed RT-qPCR in embryonic and early postnatal cerebellar tissue.

Genetic transcription varies greatly during development, hindering the identification of housekeeping genes that remain stable across distinct developmental periods (Xu et al., 2017). Thus, based on available literature and inter-plate stability, we selected different housekeeping gene pairs for each time point analyzed: *Sdha* and *Ywhaz* for E15 (Cheung et al., 2017; Xu et al., 2017), *Gusb* and *Sdha* for E18 (Cheung et al., 2017; Xu et al., 2017), and *Gusb* and *Ywhaz* for P1 and P7 (Xu et al., 2017; Shaydurov et al., 2018). Within each developmental time point, we compared the relative gene expression across WT and *Tsc1*^+/–^ genotypes.

We focused on two selected groups of genes of interest, aimed at probing the mTOR pathway and the development of different cerebellar neuronal populations. Since we used the *Tsc1*^+/–^ model, and given the fact that *Tsc1* and *Tsc2* are highly expressed in the cerebellum (Li et al., 2018), we selected these two genes for analysis. Downstream of *Tsc1*, we focused on *Mtor*, *Rictor* and *Rps6kb1*, previously shown to be selectively elevated in cerebellar tissue (Li et al., 2018). As mTOR can be found in two complexes, mTORC1 and mTORC2, in addition to *Rictor* (mTORC2), we also included *Raptor* (mTORC1) in the analysis (Sun et al., 2015). Finally, given the cerebellar enrichment for *Rps6kb1*, we also included the gene of its main protein substrate, *Rps6*, in the analysis (Mainwaring and Kenney, 2011). We then compared cerebellar development across the two genotypes, by targeting genes representative of the main cell types in the cerebellum. *Pax2* was used to investigate populations of inhibitory interneuron precursors (Maricich and Herrup, 1999), *Pax6* to assess granule cell development (Engelkamp et al., 1999), *Calb1* for Purkinje cells (Iacopino et al., 1990), and *Slc1a3* (also known as *Glast* or *Eaat1*) and *Gdf10* for astrocytes, particularly the Bergmann glia cells (Lehre and Danbolt, 1998; Cerrato et al., 2018).

**FIGURE 1:**
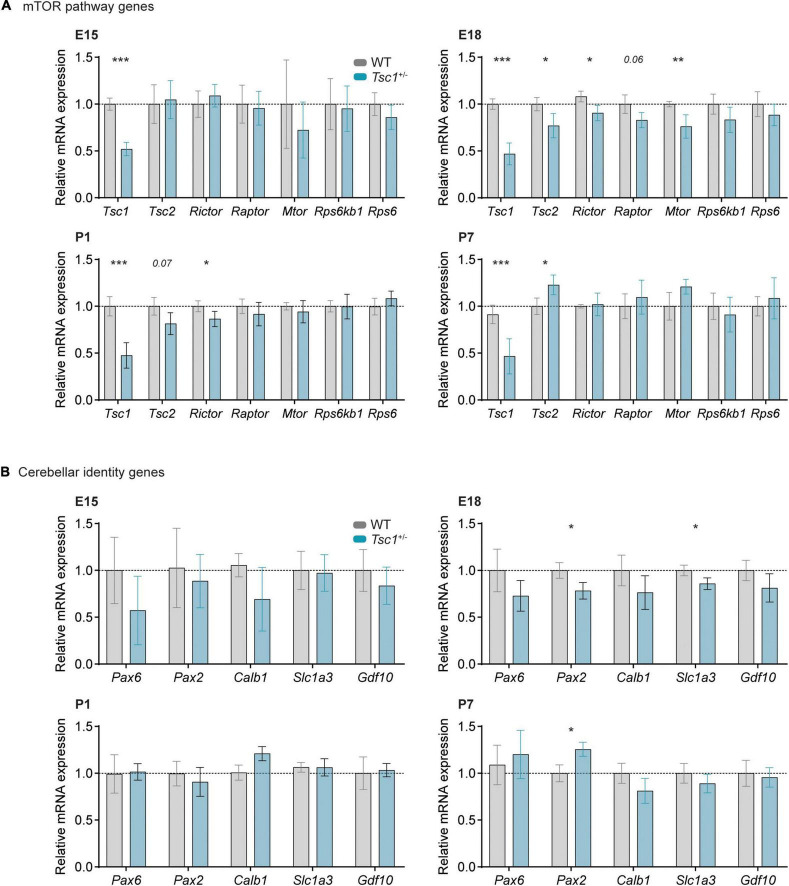
Relative gene expression is altered in *Tsc1*^+/–^ cerebella. Relative mRNA expression of mTOR pathway **(A)** and cerebellar cell type-specific genes **(B)** at E15, E18, P1 and P7. **(A)** A switch in mTOR-related gene expression occurs at E18, a time point when most markers analyzed were significantly down-regulated in *Tsc1*^+/–^ cerebella. **(B)** Relative gene expression of cerebellar identity genes is relatively stable between genotypes, with the exception of paired box gene 2 (*Pax2*) and *Slc1a3*. *t*-test, * *p* < 0.05, ** *p* < 0.01, *** *p* < 0.001; *n* = 8 mice per genotype, except for E18, where *n* = 6.

Regarding the transcription of mTOR pathway genes, we found no differences between genotypes at E15, except for the expected down-regulation of *Tsc1* transcription (*t*(13) = 8.685, *p* < 0.000001). At E18, in addition to *Tsc1* (*t*(10) = 9.320, *p* = 0.000003), also the transcription of *Tsc2* (*t*(10) = 2.389, *p* = 0.038), *Rictor* (*t*(9) = 2.521, *p* = 0.033) and *Mtor* (*t*(10) = 3.265, *p* = 0.009) was decreased in *Tsc1*^+/–^ cerebella, suggesting the presence of down-regulation mechanisms regarding mTOR complex genes. At P1, *Tsc1* (*t*(14) = 5.974, *p* = 0.000034) and *Rictor* (*t*(14) = 2.171, *p* = 0.048) were still down-regulated while, at P7, only *Tsc1* (*t*(13) = 5.341, *p* = 0.0001) and *Tsc2* (*t*(13) = 2.218, *p* = 0.045) were different from controls (Figure 1A).

We then analyzed the transcription of genes involved in the specification of distinct cerebellar cell types. No difference between genotypes was detected at E15 nor at P1. However, at E18, we found that both *Pax2* (*t*(10) = 2.867, *p* = 0.017), a marker for developing interneurons, and *Slc1a3* (*t*(10) = 2.561, *p* = 0.028), a marker for Bergman glia, were down-regulated in *Tsc1*^+/–^ cerebella, while an up-regulation of *Pax2* (*t*(13) = 2.642, *p* = 0.020) was detected in P7 *Tsc1*^+/–^ cerebellar samples (Figure 1B). These data indicate that *Tsc1*^+/–^ haploinsufficiency alters the relative expression of *Slc1a3* and *Pax2*, and dysregulates a number of mTOR pathway-related genes during early cerebellar development.

### *Tsc1* Haploinsufficiency Leads to Dysregulated Gene Interactions

**FIGURE 2:**
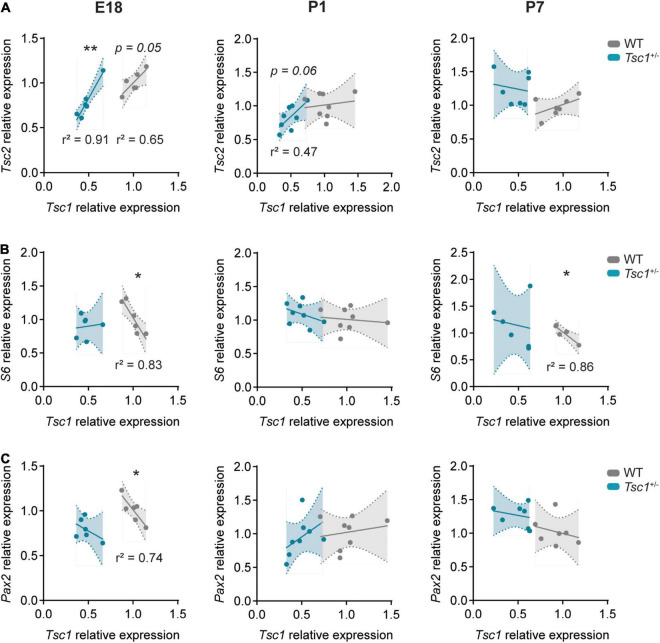
Gene correlation is lost in *Tsc1*^+/–^ cerebella. Pearson’s correlation plots for mRNA relative expression of *Tsc1* over **(A)** *Tsc2*, **(B)** *S6*, and **(C)** *Pax2* in E18 (left column), P1 (middle column) and P7 (right column) cerebella. Pearson’s correlation, * *p* < 0.05, ** *p* < 0.01; *n* = 8 mice per genotype, except for E18, where *n* = 6.

Given that E18 and P7 developmental time points presented with the largest differences between WT and *Tsc1*^+/–^ cerebellar gene expression, we focused on the E18, P1 and P7 time points, to evaluate the correlation between the relative expression of genes of interest. We found a significant positive correlation between the relative expression of *Tsc1* and *Tsc2* in WT E18 cerebella (*r*^2^ = 0.65, *p* = 0.05). While this correlation was still present in *Tsc1*^+/–^ mice at E18 (*r*^2^ = 0.91, *p* = 0.003), by P7 this relation was lost in both WT and mutants (P1: *r*^2^ = 0.02, *p* = 0.71 for WT and *r*^2^ = 0.47, *p* = 0.06 for *Tsc1*^+/–^; P7: *r*^2^ = 0.27, *p* = 0.2 for WT and *r*^2^ = 0.03, *p* = 0.72 for *Tsc1*^+/–^) (Figure 2A). Additionally, while the relative expression of *Tsc1* in WT was negatively correlated with the relative expression of *S6* (E18: *r*^2^ = 0.83, *p* = 0.011; P7: *r*^2^ = 0.64, *p* = 0.03), this was not the case for *Tsc1*^+/–^ mice (E18: *r*^2^ = 0.01, *p* = 0.82; P7: *r*^2^ = 0.03, *p* = 0.71) (Figure 2B). This indicates that genetic mTOR pathway dysregulation in *Tsc1*^+/–^ cerebella can be found early in development, and that these deficits exhibit time-dependent progression.

Paired box gene 2 expression is initiated in inhibitory interneuronal precursors during their last mitosis (Maricich and Herrup, 1999). Possibly reflecting the positive role of the mTOR pathway on neuronal differentiation (Lee et al., 2016), we found a negative correlation between *Tsc1* and *Pax2* relative expression in E18 WT cerebella (*r*^2^ = 0.74, *p* = 0.03). However, this correlation was absent in *Tsc1*^+/–^ mice (*r*^2^ = 0.22, *p* = 0.35) and lost in WT mice over time (Figure 2C). Altogether, these data suggest that mTOR pathway disruption, through *Tsc1* haploinsufficiency, could lead to early dysfunction of *Pax2*^+^ cell populations.

### *Tsc1* Haploinsufficiency Leads to Increased mTOR Signaling in the Developing Brain

Given the high prevalence of cortical malformations and epilepsy in TSC, a large number of reports has understandably used cortical and hippocampal tissue to study the possible effects of mTOR overactivation on brain morphology and pathology (Ehninger et al., 2008; Zeng et al., 2011). Here, to investigate whether mTOR signaling is also affected in the developing cerebellum at a protein level, we quantified the levels of phosphorylated S6 (Ser235/236) (pS6) at distinct developmental timepoints.

**FIGURE 3:**
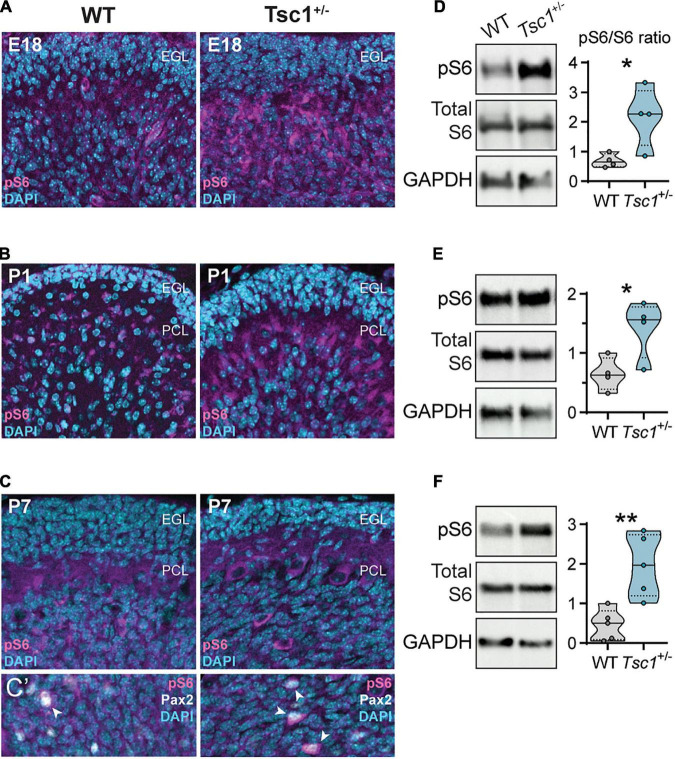
Increased cerebellar S6 phosphorylation in developing *Tsc1*^+/–^ mice. **(A–C)** Representative sagittal sections of WT and *Tsc1*^+/–^ cerebella, at E18, P1 and P7, with pS6 (Ser 235/236) labeling in magenta and DAPI in cyan. **(C’)** Detail depicting the colocalization of pS6 (Ser 235/236) (magenta) and Pax2 (white) (arrowheads). **(D–F)** Representative western blot bands for pS6 (Ser 235/236), total S6 and GAPDH, and the respective densitometry quantification normalized to WT levels. * *p* < 0.05, ** *p* < 0.01; *n* = 4 mice per genotype, except for P7, where *n* = 5.

Across the three stages of cerebellar development that we analyzed (E18, P1 and P7), we found an increase in the pS6/total S6 ratio in whole cerebella tissue samples of *Tsc1*^+/–^ mice (E18: *t*(6) = 2.88, *p* = 0.03; P1: *t*(6) = 2.77, *p* = 0.03; P7: *t*(8) = 3.85, *p* = 0.005) (Figures 3D–F). The intensity of pS6 staining was particularly visible under the external granular layer (Figures 3A–C). However, while in wild-type adults pS6 seems to be restricted to Purkinje cells (Bhatia et al., 2009), in the developing cerebellum, we also found a number of cells positive for pS6 which colocalized with Pax2 (Figure 3C’).

These findings suggest that *Tsc1* haploinsufficiency leads to cerebellar mTOR overactivation at early stages of development, associated with elevated pS6 signaling particularly in Purkinje cells and interneuronal precursors.

### *Tsc1* Haploinsufficiency Decreases Paired Box Gene 2 Expression but Does Not Affect Cell Number or Size at E18

**FIGURE 4:**
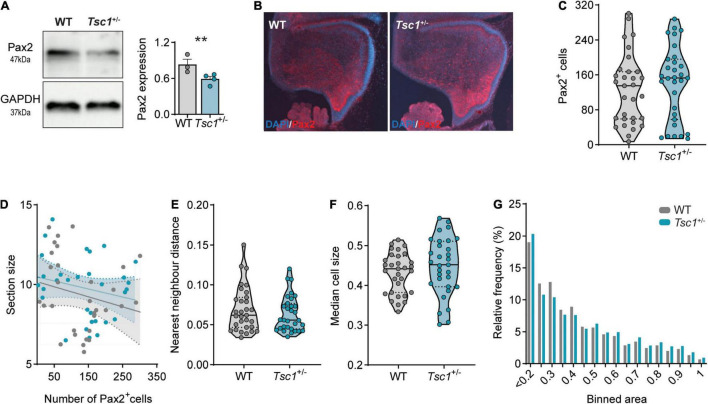
Decreased Pax2 expression in E18 *Tsc1*^+/–^ cerebella. **(A)** Representative western blot bands and quantification of Pax2 in whole cerebellar tissue (*n* = 3 WT and 4 *Tsc1*^+/–^ mice, *t*-test). **(B)** Representative Pax2-stained sagittal section (red) counterstained with DAPI (blue). **(C)** Pax2^+^ cell count of WT and *Tsc1*^+/–^ sagittal sections (*n* = 31 sections from 5 mice, Mann–Whitney test). **(D)** Pearson’s correlation between cerebellar section size and the number of Pax2^+^ cells (WT in gray, *Tsc1*^+/–^ in blue; 31 points per genotype). **(E)** Nearest neighbor distance of WT and *Tsc1*^+/–^ Pax2^+^ cells (*n* = 31 sections per genotype from 5 WT and 5 *Tsc1*^+/–^ mice; Mann-Whitney test). **(F–G)** Median cell size and relative frequency of Pax2^+^ cells over binned cell area. Area in inches. ** *p* < 0.01.

Having found that the mTOR pathway was overactive during cerebellar development, including in Pax2^+^ cells, we then investigated the expression of the latter at different developmental stages. The expression of *Pax2* in the cerebellar neurons is initiated at around E13.5, primarily in Golgi cells, and it continues into postnatal time points, with the differentiation of stellate and basket cells perinatally (Leto et al., 2006; Hoshino, 2012; Brown et al., 2019). Given that the relative mRNA expression of *Pax2* was down-regulated in *Tsc1*^+/–^ cerebella at E18, we then investigated the protein expression of Pax2 at this age. Using western blot in whole cerebellar extracts, we found that Pax2 protein was also downregulated in *Tsc1*^+/–^ cerebella (*t*(5) = 2.72, *p* = 0.04) (Figure 4A), while the number of Pax2^+^ cells in both genotypes remained similar (*U* = 426, *p* = 0.45) (Figures 4B,C).

At this stage, Pax2^+^ cells are highly migratory and quasi-uniformly dispersed through the developing cerebellum (Maricich and Herrup, 1999). We corroborated this uniform positioning in both WT and *Tsc1*^+/–^ sections as, in the two genotypes, the total number of Pax2^+^ cells was independent of the correspondent section size (WT: *r*^2^ = 0.05, *p* = 0.24; *Tsc1*^+/–^: *r*^2^ = 0.05, *p* = 0.24) (Figure 4D). To investigate the position of these cells, we then calculated the nearest neighbor distance between Pax2^+^ cells, and used this measure as a proxy for cell migration. We found that, both in WT and *Tsc1*^+/–^ mice, Pax2^+^ cells were separated by similar distances (*U* = 441, *p* = 0.58) (Figure 4E). This suggests that, while *Tsc1* haploinsufficiency causes a reduction in *Pax2* transcription and expression, this is not sufficient to affect the generation nor migration of Pax2^+^ cells at this stage in development.

Loss of function of *Tsc1*, and consequent mTOR overactivation, is often accompanied by changes in neuronal cell size (Tavazoie et al., 2005). Thus, we then measured the size of Pax2^+^ cells in the E18 cerebellum. Despite the previously reported increase in the levels of pS6 (Ser235/236)/total S6 in whole cerebellar extracts (Figures 3A,D), we found no change in the size of Pax2^+^ cells when comparing WT and *Tsc1*^+/–^ cerebella (*U* = 400, *p* = 0.26) (Figure 4F). Additionally, Pax2^+^ cells of both genotypes were present across the full range of measured cell sizes (Figure 4G). This data suggests that mTOR pathway overactivation in *Tsc1*^+/–^ E18 cerebellum leads to a decrease in Pax2 expression, without affecting the number of interneuronal progenitor cells nor their cell size.

### *Tsc1* Haploinsufficiency Leads to Perturbed Interneuron Development in P7 *Tsc1*^+/–^ Mice

**FIGURE 5:**
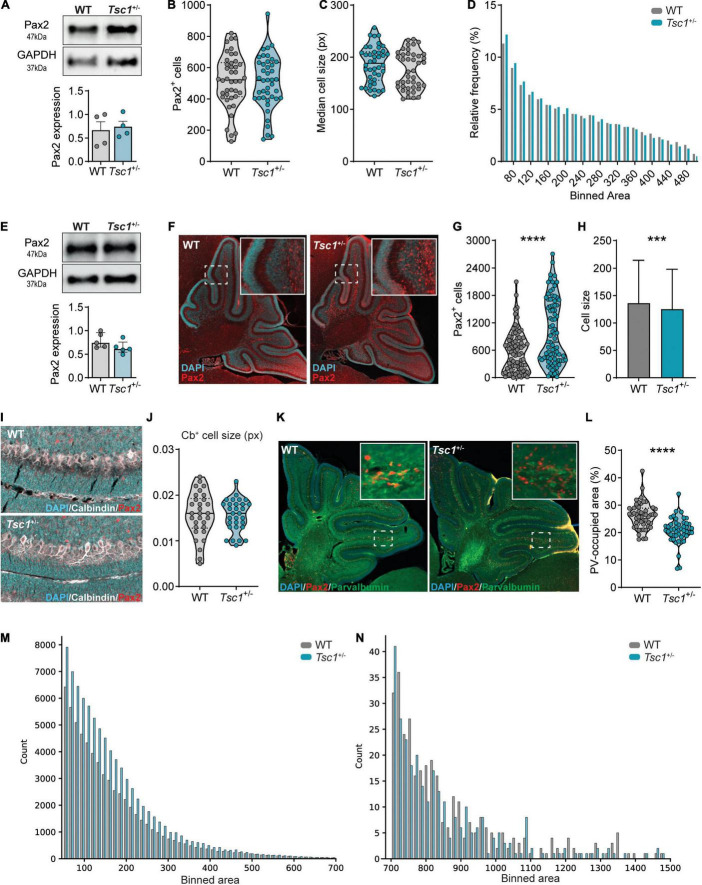
*Tsc1*^+/–^ mice present with interneuron development and maturation deficits at P7. **(A)** Representative western blot and quantification of Pax2 in P1 whole cerebellar tissue (*n* = 4 mice per genotype; *t*-test). **(B)** Pax2^+^ cell count on P1 WT and *Tsc1*^+/–^ sagittal sections (*n* = 42 sections, 4 mice per genotype, *t-*test). **(C,D)** Median cell size and relative cell frequency distribution over area (*n* = 42 sections, 4 mice per genotype, Mann–Whitney test; 22131 WT cells and 21102 *Tsc1*^+/–^ cells). **(E)** Representative western blot and quantification of Pax2 in P7 whole cerebellar tissue (*n* = 5 mice per genotype). **(F)** Representative Pax2-stained sagittal section (red) counterstained with DAPI (cyan). **(G,H)** Pax2^+^ cell count and size on P7 sagittal sections (*n* = 84 sections, 5 WT and 4 *Tsc1*^+/–^ mice, Mann–Whitney test). **(I,J)** Representative sagittal section stained for Calbindin (white), Pax2 (red) and counterstained with DAPI (cyan), with the median cell size of WT and *Tsc1*^+/–^ Purkinje cells, measured between lobules V and VI (*n* = 10 cells per mouse, 3 mice per genotype; Mann-Whitney test). **(K,L)** Representative section stained for PV (green), Pax2 (red) and Dapi (blue) and percentage of PV-occupied area per section (*n* = 51 sections from 3 WT mice and *n* = 49 sections from 3 *Tsc1*^+/–^ mice; Mann-Whitney test). **(M,N)** Frequency of small (left) and large (right) Pax2^+^ cells over binned cell area (WT = 64 058 cells *Tsc1^+/–^* = 83 369 cells). Area in pixels (px). *** *p* < 0.001, **** *p* < 0.0001.

Next, we analyzed Pax2 expression during selected postnatal timepoints. In accordance with our qPCR data, we found that Pax2 expression at P1 was similar between genotypes (Figure 5A), suggesting a normalization of the previously detected delay at E18. At E18, we found that, although Pax2 expression was decreased in *Tsc1*^+/–^ cerebella, the number of cells expressing this marker was unaltered. This remained true at P1, as we found no change in the number of Pax2 expressing cells (Figure 5B). These Pax2^+^ cells were also of similar size and distributed over the same area between genotypes (Figures 5C,D). These data are consistent with previous reports predicting small variations of Pax2^+^ cell numbers between E18 and P3 (Weisheit et al., 2006).

To further explore the increase in *Pax2* relative expression we previously found in *Tsc1*^+/–^ cerebella at P7, we next analyzed the expression and distribution of Pax2^+^ cells in the P7 cerebellum. We did not detect differences in Pax2 protein expression between WT and *Tsc1*^+/–^ cerebella (*t*(8) = 1.61, *p* = 0.76) (Figure 5E). However, in line with the qPCR data, immunohistochemical analysis of Pax2-labeled cerebellar sections revealed that *Tsc1*^+/–^ cerebella presented with an increased number Pax2^+^ cells (Figures 5F,G). Additionally, the size of these Pax2^+^ cells was smaller in *Tsc1*^+/–^ mice when compared to WT (*p* < 0.0001) (Figure 5H). This Pax2^+^ cell size change appeared to be specific toward Pax2^+^ cells, as we found no difference in the size of other cells, such as Purkinje cells (*t*(58) = 0.1219, *p* = 0.90) (Figures 5I,J).

The overall increase in pS6 (Ser235/236)/total S6 in whole cerebellar extracts suggests mTOR pathway hyperactivation, which is often accompanied by increments in cell size (Figures 3C,F). Thus, we further investigated this Pax2^+^ cell size reduction by analyzing their frequency distribution across distinct binned areas. We found that *Tsc1*^+/–^ mice presented with an increased count of small Pax2^+^ cells (Figure 5M). These small cells likely represent developing stellate and basket cells from the cerebellar molecular layer (Maricich and Herrup, 1999). Contrarily, WT and *Tsc1*^+/–^ exhibited similar cell counts for larger Pax2^+^ cells, putative Golgi cells (Simat et al., 2007; Ruigrok et al., 2011; Figure 5N). Together with the fact that no change in Pax2^+^ cell number was found at E18 and P1, these data suggest that *Tsc1* haploinsufficiency differentially affects Pax2^+^-derived lineages, leading to an increased number of Pax2^+^ stellate and basket cells.

### Maturation of Interneuron Precursors at P7 Is Altered in *Tsc1*^+/–^ Cerebella

The density of cerebellar interneurons remains relatively constant between P5 to P10 (Yamanaka et al., 2004). For the majority of these cells, which comprise molecular layer interneurons, down-regulation of Pax2 is accompanied by up-regulation of PV, a process that marks interneuron maturation (Simat et al., 2007; Grimaldi et al., 2009). Given that we found an increase in the number of Pax2^+^ cells in *Tsc1*^+/–^ cerebella at P7 but not at E18 nor P1, we hypothesized that this could also reflect a delay in the initiation of *Pax2* down-regulation in molecular layer interneurons (Weisheit et al., 2006). Therefore, we focused on the maturation of the interneuronal cells. As a proxy of cell maturation, we quantified the percentage of cerebellar area at P7, which is occupied by PV staining (*see methods*) (Figure 5K). We found that *Tsc1*^+/–^ cerebellar sections presented with a decreased percentage of surface area labeled by PV staining when compared to WT mice (median percentage: 26.45% for WT vs 20.89% for *Tsc1^+/–^, p* < 0.0001), suggesting that *Tsc1* haploinsufficiency is accompanied by deficits in the maturation of molecular layer interneurons (Figure 5L).

## Discussion

The mTOR pathway has been linked to many cellular and metabolic events, including cell replication, growth and biomass production (Liu and Sabatini, 2020). Although the mTOR kinase is known to be required for the formation of the central nervous system (CNS) (Hartman et al., 2013; Ka et al., 2014), its precise role in the development of distinct cell lineages is not completely understood. Currently, a tight regulation of the timing of mTOR pathway activation appears to be essential for the balance between undifferentiated cell proliferation and cell differentiation. In postnatal mice, mTOR signaling is detected in proliferating neural stem cells (NSCs), while knockdown of its activity reduces proliferation. Conversely, increasing mTOR activation leads to a higher number of terminally differentiated NSCs at the expense of their renewal (Hartman et al., 2013). Further, in cortical interneuron progenitors, deletion of mTOR decreases proliferation, leading to a reduction of mature calbindin-positive cells (Ka et al., 2017).

In the developing cerebellum, proper mTOR signaling appears imperative for the correct development of Purkinje cells (PC), as disruptions in either mTORC1 or mTORC2 signaling in PC from E17.5 lead to smaller soma size and deficits in dendritic arborization (Thomanetz et al., 2013; Angliker et al., 2015). Conditional deletion of *Rictor*, essential for mTORC2 kinase activity, from all CNS precursor cells at E10.5, induces early postnatal changes to PC, including the emergence of several primary dendrites and abnormal vermal macrostructure (Thomanetz et al., 2013). Furthermore, while the loss of *Rictor* does not seem to affect the development of cerebellar granule neuron precursor cells (GNP), deleting *Raptor*, necessary for mTORC1 activity, leads to a decrease in cell number (Wu et al., 2017). Additionally, increased S6 kinase activity leads to a reduction in proliferating GNP due to premature cell cycle exit (Mainwaring and Kenney, 2011).

To better understand how global mTOR overactivation impacts embryonic and early postnatal cerebellar development, we used *Tsc1*^+/–^ mice, often used as a mouse model to study mTORopathy-associated ASD. Because the mTOR kinase is an important regulator of translation (Liu and Sabatini, 2020), we first evaluated expression levels of mTOR pathway-related and cerebellar cell-specific genes in the developing cerebellum. The overall loss of correlation in distinct gene pairs we found in *Tsc1*^+/–^ mice supports a deficient stability of the TSC1-TSC2 complex, as well as a dysregulation of translational machinery. This is in line with previous work demonstrating that increased mTOR function leads to an altered profile in neuronal genetic transcription (Kim et al., 2019). Thus, *Tsc1*^+/–^ haploinsufficiency alters the translational landscape of the cerebellum early in development, through the dysregulation of central mTOR-sensitive genes.

To identify which cell types could potentially be more susceptible to mTOR overactivation, we analyzed the relative expression of cerebellar-specific cell markers in the developing cerebellum. We found cerebellar lineage deficits as early as E18, which were further evident in the first week of postnatal development. Specifically, we found that cerebellar interneuronal precursors, characterized by the expression of *Pax2*, seemed to be particularly sensitive to global haploinsufficiency of *Tsc1* and consequent mTOR pathway overactivation. Additionally, this overactivation appeared to differentially affect the development of molecular and granular layer interneurons. In fact, the observed changes in *Pax2* expression suggest that *Tsc1* haploinsufficiency leads to a delay in the initiation of Pax2 expression in embryonic development, causing its later over-compensation and altered expression pattern during later postnatal periods. At P7, although the vast majority of cerebellar interneurons is already generated, these can be found in distinct developmental stages, from migrating to fully mature neurons (Leto et al., 2006; Glassmann et al., 2009). As the changes in Pax2 expressing cells seem to be more prevalent at P7, this suggests that the numbers of Golgi cells, primarily formed prenatally, are unaffected. In contrast, the increase in Pax2^+^ cells at P7 suggests an increase in the number of stellate and basket cells. Thus, it is possible that the deficient down-regulation of mTOR signaling found in P7 *Tsc1*^+/–^ mice could contribute to an increase in cell proliferation, leading to elevated overall numbers of Pax2^+^ cells. Alternatively, disruption of mTOR signaling could also affect the timing of interneuron maturation. Nonetheless, given that Golgi cells maintain the expression of Pax2 into adulthood (Leto et al., 2006), we cannot exclude that this population is also affected by *Tsc1* haploinsufficiency. Additional studies, in older mice and with more specific interneuronal markers, could help elucidate whether changes to the total number of interneurons and their maturation status are indeed cell-specific and long-lasting.

Although the molecular mechanisms linking *Tsc1* haploinsufficiency with Pax2 dysregulation are still unclear, recent work in *Drosophila* has demonstrated direct Pax2 expression modulation by the mTOR pathway. In this model, D-Pax2 is a main regulator of cell fate in the developing eye (Avet-Rochex et al., 2014), and was shown to physically interact with the conserved zinc finger/RING domain protein, Unkempt (Unk) (Vinsland et al., 2021). In *Drosophila*, Unk expression is negatively regulated by the mTOR pathway and *Tsc1* mutants present with increased D-Pax2 expression (Avet-Rochex et al., 2014; Maierbrugger et al., 2020). It is therefore possible that the *Tsc1* haploinsufficiency leads to increased mTOR signaling and would thereby promote Unk phosphorylation and its consequent negative regulation (Bateman, 2015). Because Unk negatively regulates Pax2 expression, this could then lead to an increased number of Pax2^+^ cells. However, although Unk is highly expressed in the mouse cerebellum (Vinsland et al., 2021), additional knowledge on its regulation in the mammalian brain is needed to ascertain its role in this model.

Using immunohistochemistry, we found deficits in molecular layer interneuron maturation, as evidenced by decreased PV staining in the cerebellum of *Tsc1*^+/–^ P7 mice. PV is a calcium binding protein, abundant in Purkinje cells and cerebellar molecular layer interneurons (Kosaka et al., 1993) Thus, we focused on this population as they make up the majority of cerebellar interneuronal cells. During brain development, the initiation of PV expression coincides with the expression of a number of synaptogenesis markers, such as solute carrier family 32 and GABAA receptor α1 subunit (Simat et al., 2007). Furthermore, the amount of PV expression was shown to determine presynaptic calcium dynamics in cerebellar interneurons, modulating neurotransmitter release (Simat et al., 2007). Thus, the changes in PV expression levels and timing of the interneuronal maturation that we found in *Tsc1*^+/–^ mice, could potentially lead to deficits in synaptic integration in the cerebellum (Caillard et al., 2000). Of note, cortical PV^+^ interneurons express higher basal levels of pS6 than somatostatin^+^ interneurons, with *Tsc1* deletion from the latter leading to ectopic PV expression (Malik et al., 2019), suggesting that PV^+^ interneurons may be more sensitive to mTOR pathway dysregulation than other interneuronal populations.

Behaviorally, *Tsc1* mice models present with ASD-like features, including decreased social interaction, increased repetitive behaviors and deficient reversal learning (Goorden et al., 2007; Tsai et al., 2012). This is a similar phenotype to the one found in PV knockout mice (Wöhr et al., 2015). Conversely, decreased numbers of PV positive cells are found in other models of ASD, namely *Cntnap2*^–/–^, *Shank1*^–/–^, *Shank3B*^–/–^, and *Brinp3*^–/–^ (Filice et al., 2016; Kobayashi et al., 2018; Lauber et al., 2018; Schmitz-Abe et al., 2020). Based on this evidence, a recent review by Filice et al. proposed the “Parvalbumin Hypothesis of Autism Spectrum Disorder”, in which down-regulation of parvalbumin expression leads to altered neuronal function and abnormal neurotransmitter release, in addition to increasing reactive oxygen species production and dendritic branching (Filice et al., 2020). Thus, deficits in PV could be one of the mechanisms integrating distinct high-risk mutations that lead to the development of ASD.

## Data Availability Statement

The raw data supporting the conclusions of this article will be made available by the authors, without undue reservation.

## Ethics Statement

The animal study was reviewed and approved by an independent animal ethical committee (DEC-Consult, Soest, Netherlands), as required by Dutch law, and conform to the relevant institutional regulations of the Erasmus MC and Dutch legislation on animal experimentation.

## Author Contributions

IS, AS, and AB designed the study and analysis and wrote the first draft. IS, AS, and MO performed the qPCR and histological experiments. CO executed the western blot experiments. IS, AS, MO, and CO analyzed the data. CT, MS, and AB supervised the project. All authors contributed to the article and approved the submitted version.

## Conflict of Interest

The authors declare that the research was conducted in the absence of any commercial or financial relationships that could be construed as a potential conflict of interest.

## Publisher’s Note

All claims expressed in this article are solely those of the authors and do not necessarily represent those of their affiliated organizations, or those of the publisher, the editors and the reviewers. Any product that may be evaluated in this article, or claim that may be made by its manufacturer, is not guaranteed or endorsed by the publisher.

## Footnotes

1
ncbi.nlm.nih.gov/tools/primer-blast

2
genome.ucsc.edu/cgi-bin/hgPcr

3
pga.mgh.harvard.edu/primerbank

4
blast.ncbi.nlm.nih.gov

5
https://github.com/BaduraLab
